# Association of High Risk Human Papillomavirus and Breast cancer: A UK based Study

**DOI:** 10.1038/srep43591

**Published:** 2017-02-27

**Authors:** Nadia Aziz Salman, Giles Davies, Farida Majidy, Fatima Shakir, Hilda Akinrinade, Dhayaneethie Perumal, G. Hossein Ashrafi

**Affiliations:** 1 Kingston University London, Cancer theme, School of Life Science, Pharmacy and Chemistry, SEC Faculty, Kingston upon Thames, KT12EE, London, UK; 2 Kingston Hospital- Breast Surgery Department, Kingston upon Thames, KT2 7QB, London, UK; 3 The new Victoria hospital- Breast Surgery Department, Kingston upon Thames, KT2 7EG, London, UK; 4 Kingston University London, School of Life Science, Pharmacy and Chemistry, SEC Faculty, Kingston upon Thames, KT12EE, London, UK; 5 Fatima College of Health Sciences, Institute of Applied Technology, Department of Pharmacy, UAE

## Abstract

Infection by human papillomaviruses (HPVs) has been implicated in the aetiology of a variety of cancers. Studies evaluating the presence of HPVs in breast cancer (BC) have generated considerable controversy. To date, most studies have focused on the presence of viral DNA in BC; however there are important gaps in evidencing the role of HPV persistence in the invasiveness of BC. While these studies have been conducted in several countries, none, on the presence and biological activity of high risk (HR) HPV in BC has been done in the UK. Hence, we aimed to investigate these gaps by screening a total of 110 fresh breast tissue specimens from UK patients for the presence of twelve HR-HPV types DNA using PCR and Sanger sequencing. Samples positive for HPV-DNA were screened for viral oncoprotein expression using western blot and dot blot. Data obtained showed the presence of HR-HPVs in 42% of breast tissues of which the viral activity was only confirmed in a number of invasive carcinomas (5/26). This finding, the first to report in the UK, suggests that the selective expression of viral oncoprotein in invasive cases may propose a role for HR-HPVs in the development of some types of BC.

Breast cancer is one of the main health problems worldwide, and remains a leading cause of mortality in women^1^,^2^. Over the last decade, breast cancer incidence rates have increased by around 20% worldwide and about 4% in the UK. Since the late 1970s, these incidence rates have increased by more than half (54%). According to Cancer Research UK, the UK incidence rate in 2013 was the sixth highest in Europe with 53 300 new breast cancer cases diagnosed in females^3^.

Although it is well-known that multiple risk factors are associated with breast cancer development, in most cases the initiating cause has not been identified. This has led to studies to identify new factors related to this neoplasia^1^,^4^,^5^,^6^. Infectious agents have been implicated, as either direct carcinogens or promoters. In particular, Human Papillomaviruses (HPVs) are recognised as carcinogenic agents in breast cancer in humans^4^,^7^.

The HPVs belong to a large family of common viruses that infect cutaneous and mucosal epithelial surfaces (skin, genital) and cause both benign and malignant hyperproliferative lesions^8^,^9^,^10^. Although about 90% of HPV infections are asymptomatic and are usually cleared spontaneously by the immune system within two years, albeit after a long delay period, persistence of HPV can cause progression to malignant disease in the presence of appropriate risk factors. For example, infection of the cervix with “high risk” HPV types 16 and 18 is the initiating event in 90% of cervical cancer cases^11^,^12^,^13^,^14^,^15^. Long term viral persistence is necessary for malignancy^16^, and such persistence requires avoiding immune attack and clearance. We have previously shown that, like many viruses, HPV has several ways of subverting an effective immune response which may contribute to delaying or compromising clearance of HPV infections^17^,^18^.

Regardless, while the body is working to get the infection under control, the HPV can spread through sexual means and by skin to skin contact^15^,^19^. High risk HPV types are regarded as the most important aetiological factor for cervical cancer^15^,^20^. HPVs have also been found to cause close to half of all vaginal, penile, anal, and oral cancers^19^. These findings suggest that HPV virions may be transported from the initial infection site to other organs, and may be responsible for the development of cancer in these organs.

Investigating a relationship between HPV and breast cancer is a valid hypothesis for a number of reasons. The exposure of the mammary ducts to the external environment via the nipple areola complex could provide an entry point for HPV infection. Also, most breast cancers originate from mammary duct epithelia^21^.

To date, studies on the role of HPV in breast carcinogenesis have generated considerable controversy. Di Lonardo *et al*. (1992) were the first to report the positive relationship between HPV and breast cancer, by demonstrating the presence of HPV in 29% of breast carcinoma. Since the first discovery, several studies have investigated and detected over 60% HPV in breast cancer tissue. On the contrary, opposing studies were reported the absence of HPV DNA in breast cancer and suggested it is improbable that HPV is associated with the development of breast carcinomas. Therefore, it is still not clear whether HPV is present in breast tissue and breast tumours^22^,^23^,^24^,^25^,^26^,^27^.

Despite breast cancer emerging as a major public health imperative in the UK, studies on the aetiology of breast cancer requires further attention. Given that HPV infection may be a possible risk factor in the development of the breast cancer which causes high mortality among the global female population, and in the UK, there are no studies, to our knowledge, that investigates the implication of HPV infection in breast cancer development. Therefore, this study aims to determine the presence and the expression of 12 High-risk (HR) HPV genotypes present in biopsies of normal, benign and malignant breast tissue from patients from a UK population using highly sensitive molecular biology approaches. We also correlated the expression of HPV oncoprotein in a number of invasive cases that had tested positive for HPV DNA.

## Results

### The identification of 12 high risk HPV type sequences using PCR and Sanger sequencing

The results of the breast tissue biopsy pathological diagnosis are collated in Table 1. Of the 110 specimens, 74 samples were classified as malignant (14 *in situ* carcinoma tissues, 31 invasive carcinoma and 29 invasive and *in situ* carcinoma samples). The remaining 36 samples were classified as normal or benign (21 benign tissues; 5 benign tumour tissues with previous cancer, 4 papilloma and 6 cosmetic reduction samples).

### PCR results

**Figure 1:**
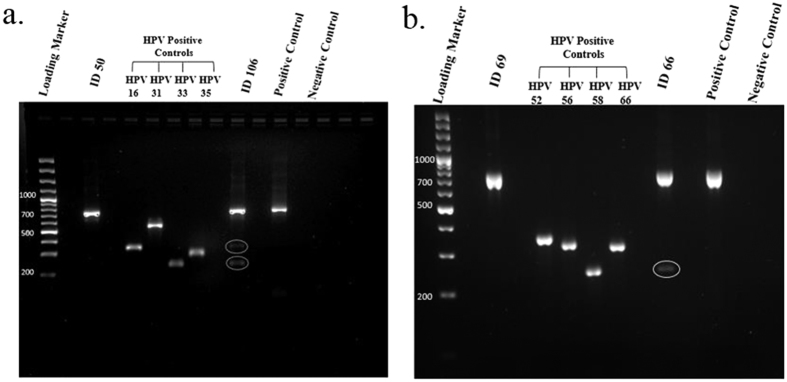
Gel electrophoresis pattern of 12 high risk HPV types by genotype specific primers amplification. (**a**) Gel electrophoresis pattern of high risk HPV types (16, 31, 33 and 35), Loading Marker = DNA ladder 100 bp plus (100 bp-3000 bp), HPV 16/35 = Positive Control DNA HPV types, 16 (325 bp), 31 (520 bp) and 33 (227 bp), 35 (280 bp) respectively, ID 50 = HPV Negative clinical sample, ID 106 = HPV Positive clinical sample, Positive Control (PC+) = Internal control; human DNA (β-globin 723 bp). (**b**) Gel electrophoresis pattern of high risk HPV types (52, 56, 58 and 66), HPV 52/66 = Positive Control DNA, HPV types 52 (360 bp), 56 (325 bp), 58(240 bp) and 66(304 bp) respectively, ID 69 = HPV Negative clinical sample, ID 66 = HPV Positive clinical sample.

Tissue specimens from a total 110 fresh abnormal breast tissues (including cancerous and non- cancerous) from women aged 25–82 years old were examined for the presence of DNA of 12 HR-HPV types using type-specific PCR. The amplification of β-globin gene (fragment size of a 723 bp) was positive in all extracted DNA, indicating an adequate quality of DNA. As a standard procedure, positive and negative controls were included for every sample run of gel electrophoresis. As shown in Fig. 1, the resulting fragments of HPV type specific positive controls and negative controls were clarified effectively and that there was no evidence of contamination, indicating a successful PCR amplification.

In each case, a and b, are representative gel electrophoresis patterns of the analysis for each set of HR-HPV types of each breast tissue sample.

### Sanger Sequencing Results

Sequencing was performed on PCR products which confirmed the amplification of HPV DNA. The BLAST analysis revealed 99% and 98% concordance of the HPV DNA sequences in breast tissue samples tested with the reference sequences for high risk HPV types. Additionally, the data obtained from the sequencing are consistent with PCR results and this further validates our findings on the detection of HPV DNA.

### Prevalence and Type distribution of HR-HPV

For each sample, the PCR experiment was repeated in triplicate to validate the accuracy of the obtained data. As shown in Tables 1 and 2, the results demonstrate that HR-HPV DNA samples were detected in 46/110 (42%) of the abnormal and normal breast cases.

In cancerous cases, HR-HPV DNA was detected with a prevalence of 47% (Table 1), in which, HPV type 39 was the most prevalent HPV genotype in 13/35 (37%) breast cancer samples, followed by types 18, 45, 16, 35 and 59 (Table 2).

Benign and normal/cosmetic reduction specimens displayed the presence of HPV in 31% (Table 1) and HPV type 16 was the most prevalent HPV genotype in 4/11 (36%) these samples, followed by types 39, 18, 45 and 59 (Table 2).

As shown in Table 3, the frequency of HPV co-infection with multiple HPV genotypes in cancerous breast tissue samples was 16/35 (46%) of cancerous cases, in which, the co-infection in invasive ductal carcinoma cases was higher with a rate of 6/16 (38%) followed by 5/16 (31%) in ductal carcinoma *in situ*.

### The expression of HPV

#### Dot Blot

**Figure 2:**
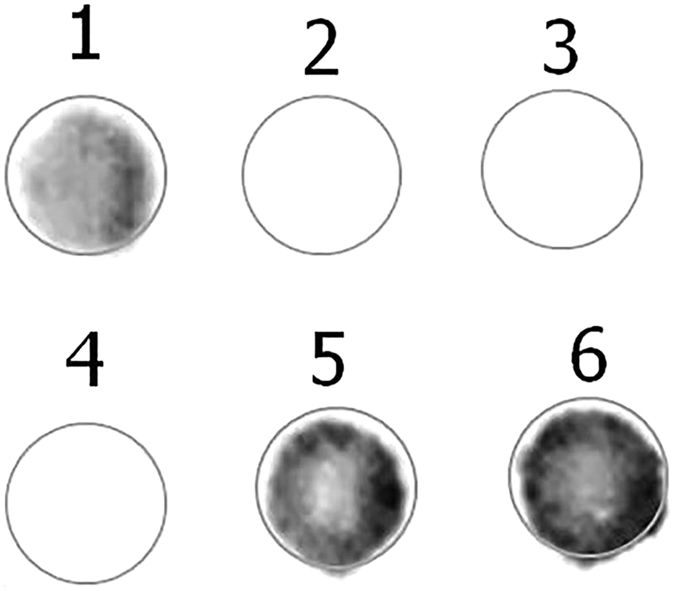
Detection of high risk HPV proteins in HPV positive samples ID 106 and 145 using Dot blot. **1** and **5** = HPV Positive representative samples ID 106 and 145 respectively, **2** and **4** = HPV Negative representative samples ID 80 and 102, **3** = negative control (HaCaT) and **6** = Positive control (CaSki).

First, we examined the expression of HPV E7 oncogenic protein that was involved in the oncogenesity of HPV using dot blot. Cell lysate from cervical cancer cell line (CaSki) that is known for expressing HPV E7 protein was used as a positive control and cell lysate from Human immortalised keratinocyte (HaCaT) cell line was used a negative control. As shown in Fig. 2, a clear precipitate was present from E7-expressing cells, positive control, while no precipitate was detected in the negative control. A clear precipitate was present from samples ID106, 120, and 145 from breast cancer samples that tested positive for HPV using PCR and sequencing.

#### Western blotting

**Figure 3:**
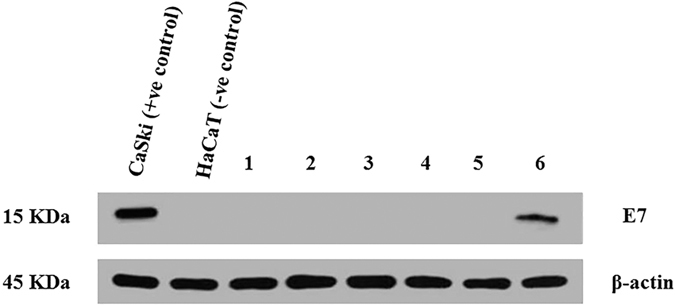
Expression of the HPV oncoprotein E7. (15 kDa) in HPV positive samples using Western blotting. β-actin expression served as a loading control (45 kDa). HaCaT and CaSki cell lines lysate served as negative and positive control respectively. 1, 2, 3 and 6 = HPV positive representative samples ID 151, 157 and 145, respectively. 4 and 5 = HPV negative representative sample ID 110 and 115 (were tested negative for HPV using PCR). Un-cropped blots are available upon request.

To complement dot blot results, we next assessed and confirmed protein expression by western blotting. Antibodies against the HPV oncoprotein E7 were used to detect the presence of viral gene expression in HPV positive breast cancer samples. As shown in Fig. 3, CaSki, cervical cancer cell used as a positive control and HaCaT cell line was used a negative control. Western blot analysis allowed the detection of E7 oncoprotein in the protein from, CaSki, cervical cancer cell line lysate as well as the protein extracted from breast cancer samples ID 145 tested HPV positive. E7 was not detected either in protein extracted from normal breast tissue or protein from breast cancer samples which were HPV negative by PCR.

In summary, this study was able to detect the presence of 12 high-risk HPV gene sequences in fresh abnormal breast tissue. The obtained results demonstrated HPV-DNA was detected in 46 out of 110 breast tissue specimens using PCR with 47% of breast cancer testing positive for HPV DNA (Table 1). Both *in situ* and invasive malignancy demonstrated the presence of HPV-DNA. Furthermore, we were able to detect HPV E7 oncogenic protein in a number of invasive breast cancer tissues which may suggest a role for HR-HPV types in the development of some type of breast cancer.

## Discussion

The global breast cancer burden in women is markedly increasing. Breast cancer is the leading cause of cancer death among women worldwide. This emphasises the importance of identifying novel etiological risk factors that are associated with breast cancer development^2^,^28^,^29^. Biological carcinogens such as viral infections play a significant role in cancer initiation since they contribute to approximately 18–20% of cancers. The lifecycle, oncogenic characteristics and molecular based evidence of HR-HPV are suggestive of a causal role for breast cancer^13^. The association of HR-HPVs with cervical cancer and various types of cancers is well established. However, the implication of HPV infection being an etiological risk factor for breast cancer carcinogenesis still remains controversial^22^.

A number of studies have investigated HR-HPV in breast cancer in which the detection of the HPV infection was based on testing for the presence of viral DNA using a range of molecular methods^30^. Since the load of HPV in breast cancer appears to be low^31^,^32^, we have in our study, applied two methods to confirm the presence of viral DNA; PCR that allowed amplification of the viral genome, and sequencing that substantially added to the validity to the outcomes.

Histopathological results on the 110 fresh breast biopsies in our study revealed that 74/110 (67%) of abnormal breast biopsies were from cancer patients (*in-situ* and invasive carcinoma) and 36/110 (33%) from benign and normal cases. The results of high-risk HPV typing showed that HPV-DNA was found in 46/110 (42%) of abnormal breast tissue specimens.

It is important to note that this study was able to detect the presence of HR-HPVs in 31% of benign and normal breast tissue specimens. This observation is consistent with the findings of other studies from different geographical areas which reported 32% and 18% of benign tissues were positive in Turkish and Australian populations, respectively^22^,^33^. However in contrast to our findings, studies conducted in France, Iran, and Switzerland failed to detect the presence of HR-HPVs in benign breast tissue specimens of the related population^23^,^24^,^34^.

The detection of HR- HPV types in benign breast samples in the UK specimens, as in the case of the Turkish and Australian studies (22, 23) calls for further investigations to fully understand the presence of HPV in these benign specimens. There exists the possibility that the benign cases may perhaps develop later to breast cancer and hence makes necessary the follow-up of the individual patients. Although we have shown the presence of HR-HPV in benign specimens, we were unable to confirm the expression of the HR-HPV protein in theses samples. While we did not find evidence of viral expression in benign cases which were positive for HPV DNA, others were able to show the expression of HPV oncoprotein in benign samples via immunohistochemistry (IHC) method^35^. The most reasonable conclusion to draw from this is that that the low level of HPV expression could be detected mainly by IHC where the viral expression can be detected even if only in a few cells. Alternatively, the absence of HPV protein could indicate that either the HPV infection has been cleared by the immune system or the viral infection is at the onset of replication which may after a long period of time persist and progress to cancer under the presence of appropriate risk factors^36^,^37^.

Our data also shows the presence of 12 HR-HPV DNA in 35/74 (47%) of breast carcinoma cases. These results validate the findings of other studies in different geographical regions worldwide including Asia and Europe, which identified the presence of high-risk HPVs in breast cancer samples. Across these studies, the presence of HR-HPVs was reported with HPV frequencies as high as 86% in some studies^38^,^39^.

In this study, the most prevalent HPV type detected in breast cancer samples positive for HPV was HPV 39 in 13 (20%), followed by HPV 18 in 8 (12%), HPV 45 in 8 (12%), and each of HPV type 16, 35 and 59 in 7 (11%) of HPV positive samples. Unlike our study, a number of reports have identified HPV types 16 and 18 as most prevalent types in breast cancer specimens, but these relied on screening a limited number of HR-HPV types^39^. This in turn limited the detection of many other HR HPV genotypes that have been detected in our study.

Additionally, examination of 12 different high-risk HPV types in each breast samples allowed for the detection of HR-HPVs coinfection in 16/35 (46%) of cancerous samples (Table 3). Multiple HR-HPV infections may interact or act synergistically to induce cancer development or could possibly lead to increase the severity of breast cancer or the risk of breast cancer progression in the presence of other cofactors. This theory supports and may possibly explain the aetiology behind the detection of multiple HPV in breast cancer pathogenesis.

Our data also revealed that the prevalence of HPVs DNA in cancerous cases was higher among women with invasive ductal carcinoma and lowest in lobular carcinoma. This observation is consistent with other reports, which highlighted the highest HPV positivity rate in patients with ductal breast carcinoma, but lower rates of HPV prevalence in other histological classifications. The presence of HPV infection in the breast ducts is a logical association as the exposure of the mammary ducts to the external environment could possibly provide an entry point for HPV infection; this may serve as cogent explanation for most breast cancers originating from the mammary duct epithelia^22^. This view is also substantiated by our findings showing a strong correlation between HR-HPV and the frequency of ductal carcinoma cases.

The detection of viral DNA has been used as a marker for the presence of the virus in related lesions, although this is not indicative of a productive infection. Hence, we investigated activity of the virus by examining the expression of HR-HPVs E7 oncoprotein using immunoblotting approaches. We were able to spot the biological activity of HR-HPVs in a number of breast cancer specimens (5/35). It is interesting to note the expression of HPV E7 oncogenic protein that is involved in the oncogenesity of HPV was only detected in invasive breast cancer specimens.

This is the first study that was able to document the expression of HR-HPV oncoprotein E7 in fresh breast cancer samples using immunoblotting techniques. We showed the expression of E7 in breast tissue from patients with breast cancer rather than in patients with early lesions or in healthy subjects. Regarding the association between the HR-HPV and tumour aggressiveness, Wazer *et al*.^40^ using human mammary epithelial cells reported that HPV E6 and E7 oncogenes were sufficient to immortalise the cells that were required for initiation and all subsequent stages of carcinogenic progression^40^. This is aligned with our findings where we detected the presence of E7 protein only in invasive ductal breast cancer cases.

HR-HPV oncogenes, E6 and E7, are expressed early in cervical carcinogenesis as they play an important role in the transformation process. Hence, these genes are expressed at accumulative levels throughout cancer progression as a result of viral DNA integration into the host genome and the subsequent effect of uncontrolled overexpression. And this ultimately leads to an abundant expression of E6 and E7 throughout the epithelia in high-grade cervical dysplasia and cervical cancer1^41^,^42^. This may also explain the higher expression of E7 oncoprotein in invasive carcinoma specimens in our study using immunoblotting methods which requires higher level of HPV proteins for detection.

However, due to the low viral load of HPV in breast cancer, we firmly believe that employing immunoblotting techniques to detect the HPV oncoprotein in breast cancer tissues can be challenging, therefore extending this investigation with further approaches such as Immunohistochemistry would be beneficial for the detection of lower levels of HPV expression.

## Conclusion

The presence of high risk HPV viral sequences and the expression of viral protein in malignant breast tissue highlight the importance of further studies to investigate and assess the possible viral mechanism in the pathogenesis of breast cancer. We have established a clear rationale for this investigation and the prospect of investigating a role of vaccination against a broader range of HR-HPV subtypes, and not just HPV type 16 and 18, in the primary prevention of breast cancer. This can provide a strong impetus to advance research in delaying disease progression or in preventative initiatives in an important global health imperative involving women.

## Materials and Methods

### Recruitment of patients and Breast tissue specimen collection

This study was formally approved by the Ethics Committee of Health Research Authority NHS (NRES Committee London-Stanmore, UK) REC reference: 12/LO/0837. All methods were carried out in accordance with the approved guidelines and regulations. Following informed consent, a total of 110 fresh breast tissue specimens were aseptically collected by one surgical team (over a period of 3 years) from patients immediately after excision in surgery in the Breast Surgery Department at Kingston Hospital, London, UK. All breast tissue specimens were formally reported by Kingston Hospital Pathology Department as part of the standard clinical diagnosis of the patients. The tumours were histopathologically classified into: benign, benign with previous cancer, *in situ* carcinoma, invasive carcinoma, and *in situ* and invasive carcinoma (Table 1).

All biopsies obtained following surgery were immediately preserved using Allprotect reagent from QIAGEN to stabilize DNA, RNA and protein, with aseptic handling of tissue in the theatre environment to avoid contamination of samples.

### DNA and Protein extraction and Purification

Separate disposable items, such as gloves, surgical blades and tubes, were used during tissue handling to avoid cross-contamination between specimens. Cellular DNA, RNA and protein were extracted from the collected breast tissue samples using Allprep DNA/RNA/Protein Mini Kit (QIAGEN) in accordance with the manufacturer’s instructions. Briefly, to obtain genomic materials and protein, this entailed the following: The tissue was cut into small pieces and then homogenised in lysis buffer using Tissue Lyser (QIAGEN) and QIAshredder spin columns. The lysate of each sample was subjected to extraction steps to obtain the purified nucleic acid as per the manufacturer’s instructions. The concentration and the purity of nucleic acids were assessed using NanoVue plus spectrometer (GE Lifesciences).

### Detection and genotyping of HPV DNA

The research approach to the identification of HPVs was to use Polymerase Chain Reaction (PCR). HPV genotyping was performed using the HPV- HCR Genotype-Eph kit (AmpliSens) using the purified DNA extracts from the 110 benign and breast cancer tissue specimens (extracted at Kingston University laboratories-UK). The kit is based on simultaneous amplification of four targeted regions of the HPV gene of each of the four HPV DNA types in one tube (multiplex-PCR). The HPV DNA amplification was run in three tubes, in which 4 different HPV types were amplified in each tube as follows; HPV-16/31/33/35, HPV-18/39/45/59 and HPV-52/56/58/66. This enabled the detection of infections and co-infections of 12 high risk HPV genotypes in each collected tissue sample.

To analyse and determine HPV genotypes, the amplified PCR products were electrophoresed along with type specific positive controls on a 3% (w/v) agarose gel stained using SYBR Safe (Invitrogen). This was then visualised under UV using a Gel Doc XR+ System (Bio-Rad). For each sample, the amplification of β-globin served as an internal positive control to determine the adequacy of the extracted DNA for amplification. PCR was performed in triplicate for each HPV positive sample to confirm the result.

### HPV DNA sequencing

In order to confirm presence of HPV genes, PCR products of the HPV positive samples were subjected to direct sequencing. All PCR products were purified using a QIAquick PCR Purification Kit (Qiagen) and then sequenced using an Applied Biosystems 3730xL analyser. The results were evaluated using NCBI BLAST program to verify HPV sequences. The use of this method was used to add to the validity of results.

### Immunoblotting

#### Dot blot

A standard dot blotting method was employed to investigate whether HR-HPVs, expressed in samples positive for HPV DNA using anti-HPV E7 monoclonal antibody (Cervimax) – Valdospan GmbH, Austria, cross reacted with a number of other HR-HPV types. Equal amounts of purified protein were directly spotted onto a nitrocellulose membrane. The membranes were blocked with 2% (w/v) bovine serum albumin (BSA) in Phosphate-buffered saline (PBS)-Tween20 (0.05% (v/v) Tween20) at room temperature for 1 hour to reduce non-specific binding. These were then washed with PBS-Tween for 5 minutes three times. The blocked membranes were incubated with E7 mouse monoclonal antibody (CERVIMAX) (diluted at 1:500 in 1% (w/v) non-fat milk in PBS-T) at 4 °C for 3–4 hours. The membranes were subjected for incubation with secondary antibody Goat Anti-Mouse IgG - Alkaline Phosphatase conjugate antibody (Invitrogen) for 2 hours at room temperature and were then washed for 5 minutes 3 times. Finally, the membranes were developed using NBT and BCIP reagent Kit (Invitrogen) for 5 minutes and then washed twice in PBS-T.

#### Western Blot

To validate the dot blot findings, the expression of HR-HPV proteins was also examined using a standard semi-dry western blotting method which was employed to investigate whether HR-HPVs, expressed in samples positive for HPV DNA using anti-HPV E7 monoclonal antibody (Cervimax) – Valdospan GmbH, Austria. Purified protein was subjected to western blot to examine the expression of HPV oncogenic protein E7. Equal amounts of protein were electrophoresed using Bolt™ 4–12% Bis-Tris gel (Invitrogen) and transferred on to 0.2 μm Nitrocelulose membrane using MES Buffer: 50 mM MES, 50 mM Tris-HCl, 0.1% (w/v) SDS, 1 mM EDTA, pH 7.3. Membranes were blocked with 5% (w/v) non-fat dry milk in TBS-T (10 mM Tris-HCl, pH 7.5, 150 mM NaCl, and 1% (v/v) Tween20) at room temperature for 2 hours to reduce non-specific binding. The membrane was then incubated with E7 mouse monoclonal antibody (CERVIMAX) (diluted at 1:500 in 1% (w/v) non-fat milk in TBS-T) at 4 °C overnight. The membrane was washed with TBS-Tween buffer for 20 minutes 3 times and then incubated with IRDye secondary antibody Donkey anti-Mouse IgG (diluted at 1:15000) (*Li-Cor*) at room temperature for 2 hours. The membrane was then visualised by OdysseyCLx Imaging System (*Li-Cor*).

## Additional Information

**How to cite this article**: Salman, N. A. *et al*. Association of High Risk Human Papillomavirus and Breast cancer: A UK based Study. *Sci. Rep.* **7**, 43591; doi: 10.1038/srep43591 (2017).

**Publisher's note:** Springer Nature remains neutral with regard to jurisdictional claims in published maps and institutional affiliations.

## Figures and Tables

**Table 1:** Identification of high risk HPV DNA in cancerous, benign and normal breast tissue specimens.

	Total Samples N (%)	HPV Positive Samples N (%)
Total Number of Samples N	110 (100)	46/110 (42)
Age (Year) (25–82)		
<50	62/110 (56)	30/46 (65)
>50	48/110 (44)	16/46 (35)
Pathological Status		
Cancerous Cases	74/74 (100)	35/74 (47)
*In Situ* Cases		
Ductal Carcinoma *In Situ* (DCIS)	13/74 (18)	8/35 (23)
Lobular Carcinoma *In Situ* (LCIS)	1/74 (1)	1/35 (3)
Invasive Cases		
Invasive Ductal Carcinoma (IDC)	23/74 (31)	10/35 (29)
Invasive Lobular Carcinoma (ILC)	8/74 (11)	5/35 (14)
Invasive and *In Situ* Cases		
Invasive & *In Situ* Ductal Carcinoma	23/74 (31)	8/35 (23)
Invasive & *In Situ* Lobular Carcinoma	6/74 (8)	3/35 (9)
Benign and Normal Cases	36/36 (100)	11/36 (31)
Benign Tissue	21/36 (58)	6/11 (55)
Benign Tissue (previous cancer)	5/36 (14)	1/11 (9)
Papilloma	4/36 (11)	3/11 (27)
Cosmetic Reduction	6/36 (17)	1/11 (9)

**Table 2:** Prevalence and type distribution of high-risk HPV genotypes in cancerous, benign and normal breast tissue specimens.

Pathological Status	HPV Genotype											
HPV Positive Cancerous Cases n = 35	16	31	33	35	18	39	45	59	52	56	58	66
*In Situ* Cases												
Ductal Carcinoma *In Situ* (DCIS) (8)	2	1	—	2	2	3	2	—	1	—	1	—
Lobular Carcinoma *In Situ* (LCIS) (1)	—	—	—	—	—	—	—	1	—	—	—	—
Invasive Cases												
Invasive Ductal Carcinoma (IDC) (10)	1	3	3	3	2	4	2	2	1	1	1	—
Invasive Lobular Carcinoma (ILC) (5)	—	1	—	1	1	2	2	1	—	—	1	—
Invasive and *In Situ* Cases												
Invasive & *In Situ* Ductal Carcinoma (8)	4	—	—	—	2	4	2	3	—	—	1	—
Invasive & *In Situ* Lobular Carcinoma (3)	—	—		1	1	—	—	—	—	—	—	—
Total Prevalence of Specific HPV Genotype Cases n =	7	5	3	7	8	13	8	7	2	1	4	—
**HPV Positive Benign and Normal Cases n = 11**	**16**	**31**	**33**	**35**	**18**	**39**	**45**	**59**	**52**	**56**	**58**	**66**
Benign Tissue (6)	2	1	—	1	—	1	1	1	—	—	2	—
Benign Tissue (previous cancer) (1)	—	—	—	—	2	—	—	—	—	—	—	—
Papilloma (3)	1	—	1	—	1	2	1	1	—	—	—	—
Cosmetic reduction (1)	1	—	—	—	—	—	—	—	—	—	—	—
Total Prevalence of Specific HPV Genotype Cases n=	4	1	1	1	3	3	2	2	—	—	2	—

(−) *negative results*.

**Table 3:** The Frequency of HPV Co-infection with Multiple HPV Genotypes in Cancerous, Benign and normal breast tissue samples.

Pathological Status	Single HPV Infection n=	Multi HPVs Co-Infection n =
Cancerous Cases (n=)		
*In Situ* Cases		
Ductal Carcinoma *In Situ* (DCIS) (8)	3	5
Lobular Carcinoma *In Situ* (LCIS) (1)	1	—
Invasive Cases		
Invasive Ductal Carcinoma (IDC) (10)	4	6
Invasive Lobular Carcinoma (ILC) (5)	3	2
Invasive and *In Situ* Cases		
Invasive & *In Situ* Ductal Carcinoma (8)	5	3
Invasive & *In Situ* Lobular Carcinoma (3)	3	—
Benign and Normal Cases (n = )		
Benign Tissue (6)	5	1
Benign Tissue (previous cancer) (1)	1	—
Papilloma (3)	1	2
Cosmetic Reduction (1)	1	—

(−) *negative results*.
